# Machine Learning Model Based on Transthoracic Bioimpedance and Heart Rate Variability for Lung Fluid Accumulation Detection: Prospective Clinical Study

**DOI:** 10.2196/18715

**Published:** 2020-08-27

**Authors:** Natasa Reljin, Hugo F Posada-Quintero, Caitlin Eaton-Robb, Sophia Binici, Emily Ensom, Eric Ding, Anna Hayes, Jarno Riistama, Chad Darling, David McManus, Ki H Chon

**Affiliations:** 1 Department of Biomedical Engineering, University of Connecticut Mansfield, CT United States; 2 Department of Medicine, University of Massachusetts Medical School Worcester, MA United States; 3 University of Massachusetts Memorial Hospital Care Worcester, MA United States; 4 Philips Research Eindhoven The Netherlands; 5 Department of Emergency Medicine, University of Massachusetts Medical School Worcester, MA United States

**Keywords:** heart failure, transthoracic bioimpedance, heart rate variability, fluid accumulation, autonomic nervous system, machine learning, cardiology

## Abstract

**Background:**

Accumulation of excess body fluid and autonomic dysregulation are clinically important characteristics of acute decompensated heart failure. We hypothesized that transthoracic bioimpedance, a noninvasive, simple method for measuring fluid retention in lungs, and heart rate variability, an assessment of autonomic function, can be used for detection of fluid accumulation in patients with acute decompensated heart failure.

**Objective:**

We aimed to evaluate the performance of transthoracic bioimpedance and heart rate variability parameters obtained using a fluid accumulation vest with carbon black–polydimethylsiloxane dry electrodes in a prospective clinical study (System for Heart Failure Identification Using an External Lung Fluid Device; SHIELD).

**Methods:**

We computed 15 parameters: 8 were calculated from the model to fit Cole-Cole plots from transthoracic bioimpedance measurements (extracellular, intracellular, intracellular-extracellular difference, and intracellular-extracellular parallel circuit resistances as well as fitting error, resonance frequency, tissue heterogeneity, and cellular membrane capacitance), and 7 were based on linear (mean heart rate, low-frequency components of heart rate variability, high-frequency components of heart rate variability, normalized low-frequency components of heart rate variability, normalized high-frequency components of heart rate variability) and nonlinear (principal dynamic mode index of sympathetic function, and principal dynamic mode index of parasympathetic function) analysis of heart rate variability. We compared the values of these parameters between 3 participant data sets: control (n=32, patients who did not have heart failure), baseline (n=23, patients with acute decompensated heart failure taken at the time of admittance to the hospital), and discharge (n=17, patients with acute decompensated heart failure taken at the time of discharge from hospital). We used several machine learning approaches to classify participants with fluid accumulation (baseline) and without fluid accumulation (control and discharge), termed *with fluid and without fluid* groups, respectively.

**Results:**

Among the 15 parameters, 3 transthoracic bioimpedance (extracellular resistance, R_0_; difference in extracellular-intracellular resistance, R_0_ – R_∞_, and tissue heterogeneity, α) and 3 heart rate variability (high-frequency, normalized low-frequency, and normalized high-frequency components) parameters were found to be the most discriminatory between groups (patients with and patients without heart failure). R_0_ and R_0_ – R_∞_ had significantly lower values for patients with heart failure than for those without heart failure (R_0_: *P*=.006; R_0_ – R_∞_: *P*=.001), indicating that a higher volume of fluids accumulated in the lungs of patients with heart failure. A cubic support vector machine model using the 5 parameters achieved an accuracy of 92% for with fluid and without fluid group classification. The transthoracic bioimpedance parameters were related to intra- and extracellular fluid, whereas the heart rate variability parameters were mostly related to sympathetic activation.

**Conclusions:**

This is useful, for instance, for an in-home diagnostic wearable to detect fluid accumulation. Results suggest that fluid accumulation, and subsequently acute decompensated heart failure detection, could be performed using transthoracic bioimpedance and heart rate variability measurements acquired with a wearable vest.

## Introduction

Heart failure is estimated to affect more than 25 million people worldwide and over 6 million people in the United States [1-4]. Acute decompensated heart failure frequently results in hospitalization and can also increase risk for arrhythmia, stroke, and death [5,6]. The most clinically apparent features associated with acute decompensated heart failure include pulmonary or peripheral edema [5,7,8]. Several validated biomarkers for acute decompensated heart failure detection exist, including body weight, B-type natriuretic protein, invasive pulmonary pressure measurement, and intrathoracic bioimpedance from cardiac implantable devices [9]. The simplest, least costly, and most widely used measure for ambulatory patients with chronic heart failure is body weight; however, body weight monitoring is not an ideal approach, since weight change correlates poorly with acute heart failure worsening, thus limiting the impact of existing home-based heart failure management programs [10].

Transthoracic bioimpedance can measure intrathoracic volume, a surrogate biomarker of pulmonary edema [11-13]. For years, it has been applied for lung fluid abnormality detection and fluid management after heart failure [14,15]. Transthoracic bioimpedance injects a small alternating current into the tissue via electrodes and measures the voltage response. By doing so, and by using Ohm’s law, the electrical resistance of the thorax can be calculated. Higher values of resistance suggest lower volumes of fluid accumulated in the lungs, and vice versa (for a detailed technical explanation of transthoracic bioimpedance, please see the Methods section). Electrocardiographic (ECG) signals are used to compute parameters of heart rate variability [16], which has been shown to be dysregulated in patients with heart failure and provides information about the autonomic nervous system [16-18].

Traditionally, various types of electrodes have been used for transthoracic bioimpedance and ECG measurements using fluid accumulation vests: adhesive Ag-AgCl electrodes, which often result in skin irritation and are often misaligned when positioned; textile electrodes, which need to be wetted prior to every use; and recently proposed reusable carbon black–polydimethylsiloxane (PDMS) dry electrodes [19,20]. In our previous work [19,20], we showed that carbon black–PDMS electrodes could be a suitable alternative to textile electrodes for measuring transthoracic bioimpedance and ECG signals using customized fluid accumulation vests. Since these electrodes are biocompatible, do not cause skin irritations, do not need to be wetted prior to use, and show comparable results to those of textile and adhesive electrodes, we decided to use carbon black–PDMS dry electrodes.

There are several studies [12,21] that have explored bioimpedance to detect acute decompensated heart failure. Our group has shown that transthoracic bioimpedance can be measured daily with fluid accumulation vests using conventional electrodes, and a predictive algorithm analyzing daily bioimpedance parameters showed reasonable performance in predicting recurrent heart failure events, including hospitalization, diuretic uptitration, and worsening heart failure symptoms [12]. Lindholm et al [22] determined that leg bioimpedance was inversely correlated with heart failure incidence, and by combining leg bioimpedance with demographic information, they obtained accurate heart failure predictions. Sato et al [23] evaluated parameters from bioelectrical impedance analysis in participants with congenital heart disease and determined that the edema index obtained from bioelectrical impedance analysis could also be a marker for heart failure severity.

In this prospective clinical study (System for Heart Failure Identification using an External Lung Fluid Device; SHIELD) to examine the performance of transthoracic bioimpedance and heart rate variability measured using carbon black–PDMS electrodes embedded in fluid accumulation vests for detection of acute decompensated heart failure, we hypothesized that (1) participants without acute decompensated heart failure should have resistance measurements that are higher than those of participants with acute decompensated heart failure at the time of admittance to the hospital; (2) participants with acute decompensated heart failure at the time of discharge from hospital should have smaller amount of accumulated lung fluid and therefore higher resistance measurements than those of participants with acute decompensated heart failure at the time of admission; and (3) autonomic function assessed by heart rate variability would provide additional information about the dysregulation of heart failure patients, hence, it would detect acute decompensated heart failure.

## Methods

### Experimental Setup

A total of 93 hospitalized individuals were prospectively enrolled in our observational study at the University of Massachusetts Medical Center. We acquired recordings from participants with acute decompensated heart failure taken within the first few hours of hospital arrival (baseline) and taken prior to discharge from hospital (discharge). We also acquired recordings from a group of patients without acute decompensated heart failure (control). All participants gave written informed consent before participating in the study, in accordance with the Declaration of Helsinki. The protocol was approved by the institutional review board of the University of Massachusetts Memorial Hospital (docket number H00014714).

**Figure 1:**
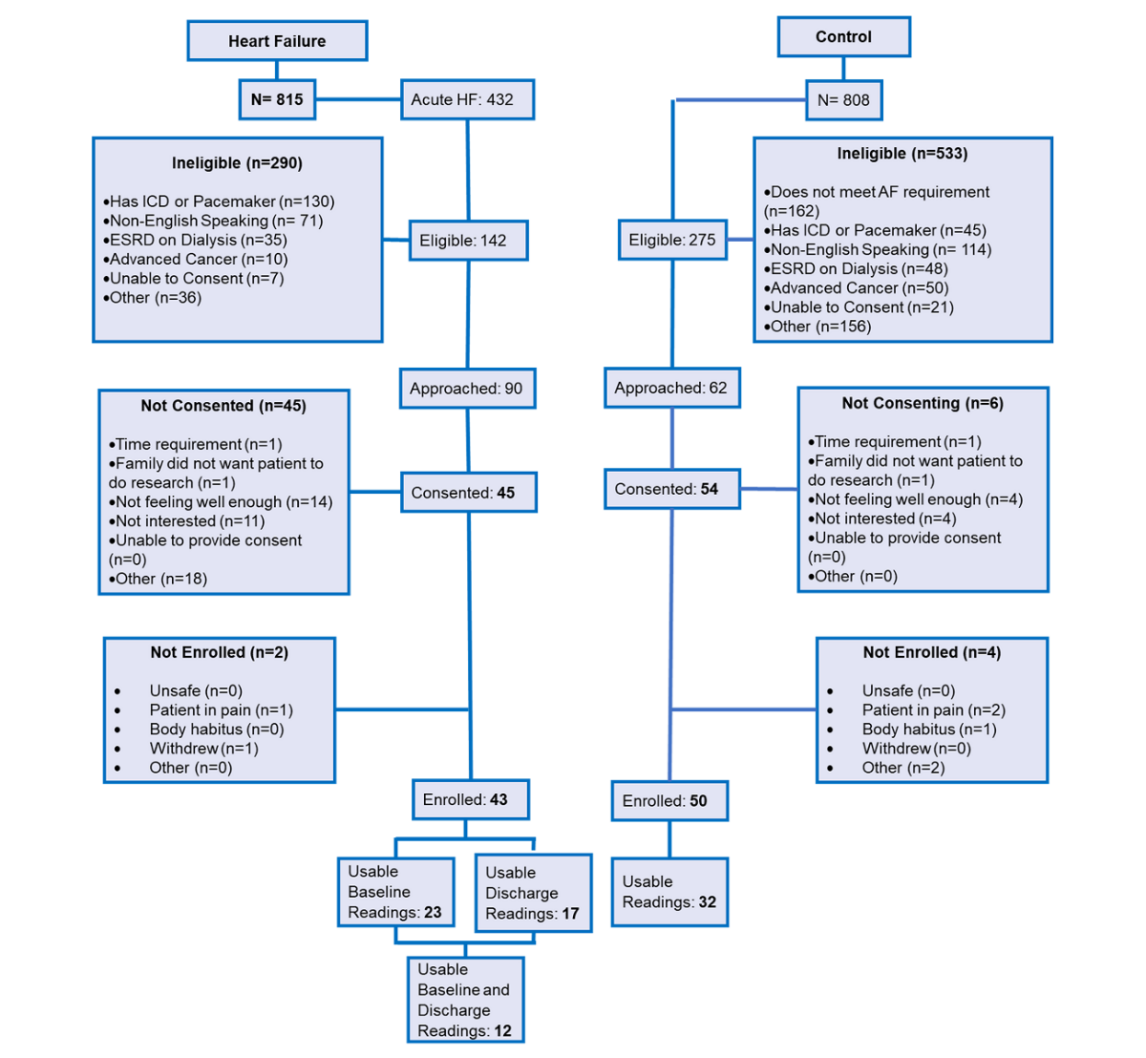
CONSORT diagram. AF: atrial fibrillation; ESRD: end-stage renal disease; HF: heart failure; ICD: implantable cardioverter-defibrillator.

The CONSORT diagram in Figure 1 depicts the screening and enrollment process for this study. We screened over 800 people for the heart failure group alone, which resulted in 432 people identified with acute heart failure. Of these 432 people, only 142 were eligible. We had strict eligibility criteria for this study. Exclusion criteria were patients with an implantable cardioverter defibrillator or pacemaker, who were non-English speaking, who were on dialysis, who had advanced cancer requiring chemotherapy, or who did not have the ability to consent. Most people were excluded from the study due to the presence of an implantable cardioverter-defibrillator or pacemaker (130/290, 44.8%). Our inclusion criteria consisted of patients who were aged over 40 years (50 years if enrolled before June 28, 2018); who were on hospital-based telemetry; who had New York Heart Association functional class II, III, or IV heart failure; and whose skin was intact.

For this study, we used Philips prototype fluid accumulation vests [12], which provide transthoracic bioimpedance measurements at 16 frequencies in the range from 10 kHz to 999 kHz and ECG recordings at 256 Hz. Participants wore the vest without clothing, so that its 4 electrodes were affixed to their left and right abdomen. Copper mesh carbon black–PDMS electrodes were used [24]. These electrodes have been proven to provide consistent transthoracic bioimpedance and ECG measurements when used with this vest [19]. For each recording, participants were asked to sit still for 10 minutes while seated on a chair with their legs resting on the floor. Once the recording was completed, a device attached to the vest wirelessly transmitted the data via a secure Bluetooth connection to a mobile phone (Samsung Galaxy Gio GT-S5660). The data were saved on an extractable secure digital memory card on the mobile phone and subsequently transferred to a PC for processing and analysis. Patients needed to be able to remain seated for at least 15 minutes to participate in the study.

### Transthoracic Bioimpedance Measurements

Transthoracic bioimpedance is a noninvasive method that measures the impedance of the tissue at a series of frequencies. A small alternating current, typically ranging from 100 µA to 10 mA, is injected into the tissue via electrodes, while the voltage drop is measured as the output. By applying Ohm’s law, the resistance of the body tissue can be calculated. Biological tissue is typically modeled with a resistance *R_0_* to represent the extracellular fluid, in parallel with a resistance *R_I_* to represent intracellular fluid, and a capacitance *C_m_* to represent cell membranes [17]. Electrical current with a frequency *f*=0 Hz will pass around all cells, and the total resistance is equal to the resistance from the extracellular fluid only, *R_0_*. At the other extreme, when the frequency is infinite, *f*=∞, the current will pass through the cells, and the total resistance can be calculated as the parallel circuit of *R_0_* and *R_I_*,

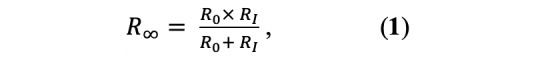



where *R_I_* can be represented as

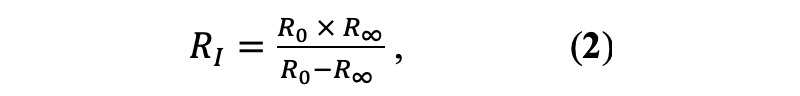



If we measure impedance for frequencies between these two extreme cases, we obtain an arc-like Cole-Cole plot in the impedance plane [25-27]. The equation for the model of the Cole-Cole plot [28,29] is

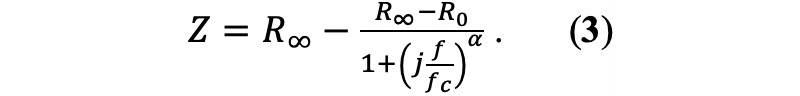



The parameters of the model can be extrapolated from a set of measurements made at a predefined set of frequencies. The exponent α represents the heterogeneity of the tissue in the model. For each frequency, the real (resistance) and imaginary (reactance) part of the electrical impedance is estimated. The Taubin algorithm [30] is used to fit a circle onto the measured impedance data. From the data computed using the Taubin algorithm, parameters of the Cole model are estimated using a heuristic search method, the Nelder-Mead algorithm [31].

**Figure 2:**
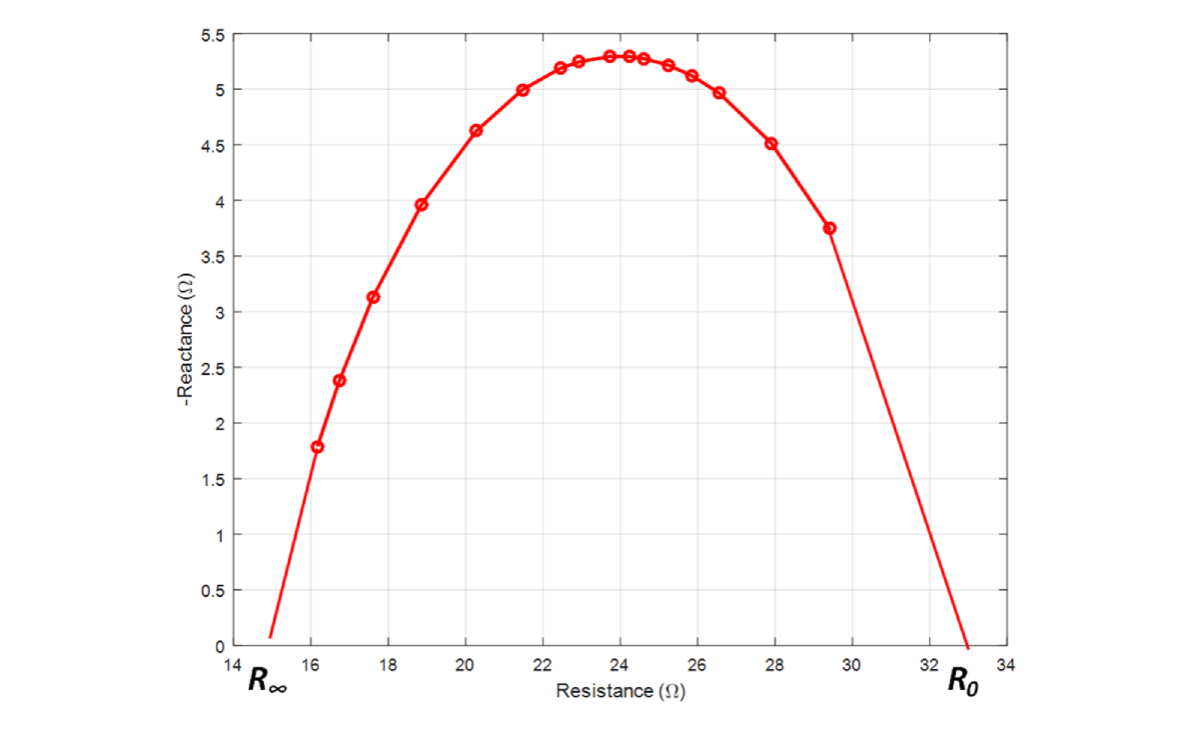
Illustrative example of the Cole-Cole plot of one patient.

Figure 2 shows an illustrative example of a Cole-Cole plot for one of the participants. The value of *R_0_* is obtained as the *x*-axis intercept at the far right side of the Cole-Cole plot, while the value of *R_∞_* is the *x*-axis intercept at the far left side of the same plot. The frequency that corresponds to the upper point of the circle is called the resonance frequency, *f_c_*,

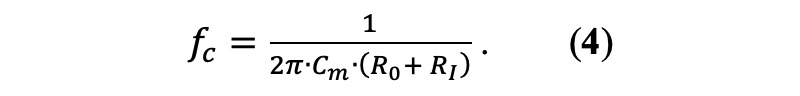



The sum of the square error is minimized in the fitting process. The *fitting error* was calculated as the sum of the square error at the optimal parameters. We calculated 8 transthoracic bioimpedance measurements in this study: *R_0_*, *R_I_*, *R_∞_*, *R_0_* – *R_∞_*, *f_c_*, *C_m_*, α, and fitting error.

### Heart Rate Variability Measurements

To compute heart rate variability parameters, 4 minutes of clean ECG data were extracted from each 5-minute recording of ECG acquired simultaneously with transthoracic bioimpedance measurements. Noise and motion artifacts were removed from the ECG signals using a bandpass filter (0.05 Hz-40 Hz). The R peaks were detected using a validated algorithm [32,33]. Segments were visually inspected to ensure correct heartbeat detection. The R-R intervals were computed, and the time series was transformed to an evenly sampled signal (sampling frequency: 4 Hz) using cubic-spline interpolation. Mean heart rate was computed as a parameter. A 256-point Blackman window was applied to each segment, and the fast Fourier transform was calculated for each windowed segment. Finally, the power spectra of the segments were averaged.

We computed the indices of low frequencies of heart rate variability (low-frequency components of heart rate variability: 0.045 Hz to 0.15 Hz), high frequencies of heart rate variability (high-frequency components of heart rate variability: 0.15 Hz to 0.4 Hz), and the indices normalized to the total power of heart rate variability (normalized low-frequency components of heart rate variability, normalized high-frequency components of heart rate variability) [16]. Indices obtained from the low-frequency components of heart rate variability represent sympathetic control, and indices from the high-frequency components of heart rate variability power represent parasympathetic control. Furthermore, we derived 2 more parameters of heart rate variability based on principal dynamic modes, a nonlinear method designed to extract only the principal dynamic components of the signal via eigendecomposition [18]. The principal dynamic mode technique is able to separate sympathetic (principal dynamic mode index of sympathetic function) and parasympathetic (principal dynamic mode index of parasympathetic function) dynamics from heart rate variability [17,18]. Table 1 includes the parameters computed in this study.

**Table 1:** Transthoracic bioimpedance and heart rate variability parameters computed in this study.

Parameter		Description
**Transthoracic bioimpedance**		
	*R*_0_	Model resistance of biological tissue—extracellular fluid or resistance when *f*=0
	*R*_I_	Model resistance of biological tissue—intracellular fluid
	*R*_∞_	Resistance of biological tissue when *f*=∞
	*R*_0_ *–R*_∞_	Range of *x* values in Cole-Cole plot
	*f*_c_	Characteristic frequency, ie, frequency corresponding to the upper point of Cole-Cole plot circle
	*C*_m_	Cell membrane capacitance
	*α*	Exponent of the model representing tissue heterogeneity
	Fitting error	Sum of squared error of the optimal Cole-Cole plot model
**Heart rate variability**		
	LF^a^ HRV^b^	Low-frequency components of heart rate variability power
	Normalized LF HRV	Normalized low-frequency components of heart rate variability power
	HF^c^ HRV	High-frequency components of heart rate variability power
	Normalized HF HRV	Normalized high-frequency components of heart rate variability power
	PDMI sympathetic^d^	Sympathetic function heart rate variability dynamics
	PDMI parasympathetic^e^	Parasympathetic function heart rate variability dynamics

^a^LF: low-frequency.

^b^HRV: heart rate variability.

^c^HF: high-frequency.

^d^PDMI sympathetic: principal dynamic mode index of sympathetic function.

^e^PDMI parasympathetic: principal dynamic mode index of parasympathetic function.

### Statistical Analysis and Machine Learning Classification

The normality of each parameter was tested using the Kolmogorov-Smirnov test [34-36]. We tested the differences in the parameters of transthoracic bioimpedance and heart rate variability between control, baseline, and discharge groups, using one-way ANOVA with Tukey posthoc for normally distributed data and the Dunn test for nonnormally distributed data (MATLAB, version 9.6; The Mathworks). The Dunn test is a nonparametric analog to multiple pairwise *t* tests following rejection of an ANOVA null hypothesis [37]. A *P* value<.05 was considered statistically significant for ANOVA and Dunn tests.

Statistical analysis of the differences between groups provides insight into the suitability of the measures of transthoracic bioimpedance and heart rate variability to detect fluid accumulation, which is used as an indication of heart failure exacerbation. However, measurement results have nonlinear characteristics and cannot be completely described with linear statistical methods. Hence, we used nonlinear methods such as machine learning to examine 15 features derived from transthoracic bioimpedance and heart rate variability for classification between groups (control, baseline, and discharge). Furthermore, participants in the discharge group were partially recovered, so they could be considered similar to the participants in control group. We tested the feasibility of classifying participants without fluid accumulation in the lung, termed *patients without fluid* (control and discharge groups) and participants with increased fluid in the lungs, termed *patients with fluid* (baseline group)

For these classification analyses, 3 algorithms were used: support vector machines [38], a *k*-nearest neighbor classifier (*k*=1) [39], and decision trees [40]. Cubic, quadratic, and Gaussian (C=1, γ=2.6) kernels were used for the support vector machine algorithm. All combinations of the 15 parameters were tested with the abovementioned classifiers to discriminate control/baseline/discharge groups, and patients with fluid/patients without fluid conditions. To compensate for the imbalance of the classes, the prior probabilities of the classes were set to be uniform in the training process. Leave-one-subject-out cross-validation was used to evaluate the performance of the machine learning models to prevent overfitting. Accuracy was computed as the number of correct classifications, divided by the total number of classifications performed, which corresponds to the number of participants in this case (N=60). Furthermore, the confusion matrices of the best models were obtained for a more detailed analysis.

## Results

We approached 90 patients with heart failure who were eligible, and 43 were enrolled in this study. Out of the 43 enrolled participants, we were able to collect data from 28 participants with heart failure; 23 were included in the baseline group (mean 72, SD 10.7 years), and 17 were included in the discharge group (mean 72.4, SD 9 years). Only 12 participants were included in both baseline and discharge groups. There were several reasons for the lower number of participants in the discharge group: (1) in some cases, the recordings were of poor quality (n=14); (2) some participants (n=5) were lost to follow-up (ie, owing to a late night or weekend discharge); (3) some participants (n=7) could not provide the second recording owing to illness or refusal.

We enrolled 50 participants without acute decompensated heart failure (mean 71.5, SD 8.5 years) in the control group. Of the recordings taken on the 50 enrolled participants 32 of them were usable. It should be noted that participants from both groups were well matched with respect to age.

The demographic and medical characteristics of study participants are shown in Table 2. There were no significant differences in the demographic characteristics of the control group compared with those of participants with heart failure (age: *P*=.70; sex: *P*=.70; race: *P*=.52). Participants with acute decompensated heart failure were more likely to have a history of cardiovascular disease risk factors (coronary artery disease: *P*=.04; myocardial infarction: *P*=.03), prior heart failure (*P*<.001), and atrial fibrillation (*P*<.001). All transthoracic bioimpedance and heart rate variability parameters were found to be normally distributed, except for low-frequency components of heart rate variability and high-frequency components of heart rate variability.

**Table 2:** Demographic and clinical characteristics.

Characteristic		Control (n=32)		Acute decompensated heart failure (n=28)		*P* value	
Age, mean (SD)		71.5 (8.5)		72.4 (10.3)		.70	
**Sex, n (%)**							
	Male		19 (59)		18 (64)		.70
	Female		13 (41)		10 (36)		
**Race, n (%)**						.52	
	White		29 (91)		26 (93)		
	Black		1 (3)		2 (7)		
	Other^a^		2 (6)		0 (0)		
Chest circumference (cm), mean (SD)		105.4 (14.1)		107.8 (13.1)		.57	
**BMI (kg/m^2^), mean (SD)**		27.7 (5.1)		29.3 (6.6)		.28	
**Medical history, n (%)**							
	Myocardial infarction		3 (9)		9 (32)		.03
	Coronary artery disease		7 (22)		13 (46)		.04
	Hypertension		20 (63)		23 (82)		.09
	Stroke/transient ischemic attack		2 (6)		3 (11)		.50
	Previous diagnosis of heart failure		1 (3)		17 (61)		<.001
	Diabetes		6 (19)		7 (25)		.56
	Dyslipidemia		23 (72)		20 (71)		.97
	Chronic lung disease		4 (13)		9 (32)		.06
	Renal failure		2 (6)		3 (11)		.53
	Atrial fibrillation		0 (0)		13 (46)		<.001
**Vital signs and serum laboratories, mean (SD)**							
	Heart rate (beats/min)		75.4 (13.2)		84.4 (25.1)		.09
	Systolic blood pressure		141.1 (28.6)		146.1 (28.7)		.51
	Diastolic blood pressure		79.9 (13.1)		81.3 (17.3)		.72
	Respiratory rate (breaths/min)		18.3 (2.2)		20.7 (2.8)		<.001
	Sodium (mg/dL)		138.8 (2.4)		138.9 (2.8)		.97
	Potassium (mg/dL)		4.1 (0.4)		4.1 (0.8)		.79
	Glucose (mg/dL)		121.6 (45.4)		143.5 (80.9)		.20
	Blood urea nitrogen (mg/dL)		19.2 (6.7)		26.3 (18.9)		.06
	Creatinine (mg/dL)		1.1 (0.4)		1.3 (0.6)		.15
	B-type natriuretic peptide^b^		112.0 (76.2)		1013.9 (1004.5)		.14
	Troponin^b^		0.2 (1.0)		0.2 (0.9)		.96
	INR		1.3 (0.7)		1.4 (0.5)		.95
**Medication use, n (%)**							
	Beta blocker		2 (6)		2 (7)		.89
	Angiotensin converting enzyme inhibitor		5 (16)		1 (4)		.12
	Diuretic		2 (6)		3 (11)		.53
	Statin		6 (19)		3 (11)		.38
	Oral anticoagulant		2 (6)		0 (0)		.18

^a^Asian; American Indian, or Alaska Native; Native Hawaiian or other Pacific Islander.

^b^Data for the control group is for 6 patients only.

We compared values of 15 parameters from transthoracic bioimpedance and heart rate variability measurements between participants in control, baseline, and discharge groups (Table 3). As can be noted, values of 2 parameters, *R_0_* and *R_0_ – R_∞_*, for the baseline group had statistically significantly lower values than those for the control group, with *P*=.006 and *P*=.001, respectively. Even though values of these 2 parameters for the discharge group were higher than those for the baseline group, there were no statistically significant differences (*R_0_*: *P*=.99; *R_0_ – R_∞_*: *P*=.57). Possible reasons could be the lower number of participants in the discharge group (discharge: n=17; baseline: n=23), and one possibility is that, at the time of discharge, some of the participants still had excess fluid in their lungs. The parameter α for the baseline group had statistically significantly higher values than those of the control group (*P*=.003),

**Table 3:** Values of transthoracic bioimpedance and heart rate variability parameters.

Parameters		Control (n=32), mean (SD)		Baseline (n=23), mean (SD)		*P* value		Discharge (n=17), mean (SD)		*P* value	
**Transthoracic bioimpedance**											
	*R*_0_ (Ω)		38.1 (10.8)		26.5 (12.8)^a^		.006		34.2 (17.4)		.99
	*R*_I_ (Ω)		52.0 (17.0)		52.0 (24.7)		>.999		54.3 (23.3)		>.999
	*C*_m_ (F)		4.08·10^–8^ (2.96·10^–8^)		4.60·10^–8^ (1.71·10^–8^)		>.999		4.42·10^–8^ (1.85·10^–8^)		>.999
	α		0.609 (0.0881)		0.716 (0.121)^a^		.003		0.646 (0.144)		.87
	*f*_c_ (Hz)		6.11·10^–4^ ( 3.45·10^–4^)		5.34·10–4 (1.51·10^–4^)		.83		5.07·10^–4^ (1.72·10^–4^)		.56
	Fitting error (Hz)		334 (669)		232 (389)		.51		347 (374)		.35
	*R*_∞_ (Ω)		21.5 (6.0)		17.0 (7.5)		.08		20.3 (9.1)		>.999
	*R*_0_*– R*_∞_ (Ω)		16.6 (6.1)		9.54 (6.0)^a^		.001		13.9 (8.8)		.57
**Heart rate variability**											
	LF^b^ HRV^c^		3.5 (4.2)		19.3 (43.4)		.06		19.2 (51.3)		.09
	Normalized LF HRV		7.4 (14.4)		32.9 (55.7)^a^		.02		34.6 (57.0)^a^		.01
	HF^d^ HRV		0.225 (0.134)		0.178 (0.092)		.38		0.127 (0.085)^a^		.01
	Normalized HF HRV		0.255 (0.154)		0.391 (0.134)^a^		.003		0.371 (0.129)^a^		.02
	PDMI sympathetic^e^		11.8 (5.52)		17.2 (12.4)		.06		15.3 (5.98)		.52
	PDMI parasympathetic^f^		13.2 (5.47)		17.1 (10.4)		.20		17.9 (7.56)		.14
	Mean heart rate		72.3 (11.9)		74.1 (18.0)		>.999		74.7 (15.9)		>.999

^a^Denotes a statistically significant difference with respect to control group.

^b^LF: low-frequency.

^c^HRV: heart rate variability.

^d^HF: high-frequency.

^e^PDMI sympathetic: principal dynamic mode index of sympathetic function.

^f^PDMI parasympathetic: principal dynamic mode index of parasympathetic function.

As for the heart rate variability parameters, for the baseline and discharge groups, high-frequency components of heart rate variability (baseline: *P*=.02; discharge: *P*=.13) and normalized high-frequency components of heart rate variability (baseline: *P*=.003, discharge: *P*=.02) had significantly higher values than those for the control group. Normalized low-frequency components of heart rate variability exhibited a significantly lower value in the discharge group, when compared to those in the control group (*P*=.01). None of the other parameters of heart rate variability exhibited significant differences between groups.

Tables 4 and 5 include the results for the machine learning classification analysis. First, only transthoracic bioimpedance parameters were used for control/baseline/discharge classification and with fluid/without fluid classification. The most accurate model for transthoracic bioimpedance parameters only for classification of control/baseline/discharge was the Gaussian support vector machine, which reached an overall accuracy of 68% using *R_0_*, *R_I_*, and α. For patients without fluid/patients with fluid classification using only transthoracic bioimpedance parameters, cubic support vector machine and gaussian support vector machine models achieved 82% accuracy, although the cubic support vector machine required less parameters (*R_0_*, α, fitting error, *R_∞_*). Incorporating the heart rate variability parameters improved the accuracy of most models. The quadratic support vector machine model achieved 75% accuracy using 8 parameters (*C_m_*, *f_c_*, fitting error, *R_∞_*, *R_0_ – R_∞_*, normalized low-frequency components of heart rate variability, normalized high-frequency components of heart rate variability, mean heart rate). As for patients without fluid/patients with fluid classification, the overall best model was the cubic support vector machine, which achieved an accuracy of 92% using 6 parameters (*R_0_*, *R_I_*, *C_m_*, low-frequency components of heart rate variability, principal dynamic mode index of parasympathetic function, mean heart rate).

Table 6 shows the confusion matrix for the most accurate model for control/baseline/discharge classification (quadratic support vector machine), and Table 7 shows the confusion matrix for the most accurate model for patients without fluid/patients with fluid classification (cubic support vector machine). In control/baseline/discharge classification, the control and baseline groups were correctly classified 78% and 83%, respectively. However, the discharge group was accurately classified only in 59% of the cases. It is worth highlighting that this group was misclassified 29% of the time as the control group. In the patients without fluid/patients with fluid classification, the patients without fluid condition (control and discharge groups) were classified correctly 96% of the time, and patients with fluid (baseline group) condition was correctly classified in 82% of the time.

**Table 4:** Highest accuracy and parameters included for control/baseline/discharge classification in each machine learning algorithm.

Type			Cubic SVM^a^	Quadratic SVM	Gaussian SVM	*k*-Nearest neighbor	Decision tree
**Transthoracic bioimpedance**							
	Accuracy, %		63	61	68	67	72
	**Parameters**						
		*R*_0_	x	x	x	x	x
		*R*_I_	x		x		
		*C*_m_		x		x	
		α			x	x	x
		*f*_c_				x	x
		Fitting error	x	x			
		*R*_∞_		x		x	
		*R*_0_ *– R*_∞_					x
**Heart rate variability**							
	Accuracy, %		58	63	56	57	53
	**Parameters**						
		LF^b^ HRV^c^	x	x			
		Normalized LF HRV	x	x	x	x	x
		HF^d^ HRV	x	x		x	x
		Normalized HF HRV		x			x
		PDMI sympathetic^e^		x		x	
		PDMI parasympathetic^f^	x	x	x	x	
		Mean heart rate		x		x	x
**Transthoracic bioimpedance and heart rate variability**							
	Accuracy, %		74	75	68	74	72
	**Parameters**						
		*R*_0_	x	x	x		x
		*R*_I_	x	x	x		
		*C*_m_	x			x	
		α	x		x	x	x
		*f*_c_		x			x
		Fitting error	x	x		x	
		*R*_∞_		x			
		*R*_0_ *– R*_∞_		x			x
		LF HRV	x				
		Normalized LF HRV					
		HF HRV	x	x		x	
		Normalized HF HRV		x			
		PDMI sympathetic				x	
		PDMI parasympathetic					
		Mean heart rate	x	x		x	

^a^SVM: support vector machine.

^b^LF: low-frequency.

^c^HRV: heart rate variability.

^d^HF: high-frequency.

^e^PDMI sympathetic: principal dynamic mode index of sympathetic function.

^f^PDMI parasympathetic: principal dynamic mode index of parasympathetic function.

**Table 5:** Highest accuracy and parameters included for patients without fluid/patients with fluid classification on each machine learning algorithm

Type			Cubic SVM	Quadratic SVM	Gaussian SVM	*k*-Nearest neighbor	Decision tree
**Transthoracic bioimpedance**							
	Accuracy, %		82	75	82	78	79
	**Parameters**						
		*R*_0_	x	x	x	x	
		*R*_I_					
		*C*_m_			x	x	
		α	x				
		*f*_c_			x		x
		Fitting error	x		x		
		*R*_∞_	x	x	x	x	x
		*R*_0_ *– R*_∞_					x
**Heart rate variability**							
	Accuracy, %		75	76	75	71	72
	**Parameters**						
		LF^b^ HRV^c^		x			
		Normalized LF HRV	x		x		x
		HF^d^ HRV		x	x	x	
		Normalized HF HRV	x				x
		PDMI sympathetic^e^	x		x	x	
		PDMI parasympathetic^f^		x			
		Mean heart rate		x	x	x	
**Transthoracic bioimpedance and heart rate variability**							
	Accuracy, %		92	88	83	85	81
	**Parameters**						
		*R*_0_	x		x	x	
		*R*_I_	x				x
		*C*_m_	x	x		x	
		α					
		*f*_c_		x			
		Fitting error			x	x	
		*R*_∞_		x	x		x
		*R*_0_ *– R*_∞_		x			
		LF HRV	x			x	
		Normalized LF HRV				x	x
		HF HRV		x	x		
		Normalized HF HRV		x		x	
		PDMI sympathetic					
		PDMI parasympathetic	x			x	
		Mean heart rate	x	x		x	

^a^SVM: support vector machine.

^b^LF: low-frequency.

^c^HRV: heart rate variability.

^d^HF: high-frequency.

^e^PDMI sympathetic: principal dynamic mode index of sympathetic function.

^f^PDMI parasympathetic: principal dynamic mode index of parasympathetic function.

**Table 6:** Confusion matrix for quadratic support vector machine—the most accurate model for control/baseline/discharge classification.

Actual	Predicted, %		
	Control	Baseline	Discharge
Control	78.1	6.3	15.6
Baseline	13.0	82.6	4.3
Discharge	29.4	11.8	58.8

**Table 7:** Confusion matrix for cubic support vector machine—the most accurate model for patients without fluid/patients with fluid classification.

Actual	Predicted, %	
	Patients with fluid	Patients without fluid
Patients with fluid	82.6	17.4
Patients without fluid	4.1	95.9

### Discussion

### Principal Findings

In this prospective observational study, we successfully trained machine learning models to classify participants with and without fluid accumulation using parameters obtained with a fluid accumulation vest, specifically transthoracic bioimpedance and heart rate variability parameters. We achieved a cross-validation accuracy of 92% using a cubic support vector machine model. The transthoracic bioimpedance parameters that contributed to this accuracy were related to intra- and extracellular fluid, whereas the heart rate variability parameters were mostly related to sympathetic activation. Our results suggest that the transthoracic bioimpedance and heart rate variability signals acquired with a wearable vest with carbon black–PDMS dry electrodes are suitable for detecting fluid accumulation and can potentially help with prediction and management of clinical worsening in heart failure patients.

In the past, transthoracic bioimpedance has been used for lung fluid abnormality detection [14,15]. In this study, we aimed to test the feasibility of a more accurate detection method for fluid accumulation by combining transthoracic bioimpedance and heart rate variability, given the autonomic dysregulation observed in heart failure patients. We used fluid accumulation vests to capture transthoracic bioimpedance and heart rate variability simultaneously. The accuracy of lung fluid abnormality detection using both transthoracic bioimpedance and heart rate variability was 92%, which is considerably higher than the maximum accuracy achieved using either only transthoracic bioimpedance (82%) or only heart rate variability (76%). Although the maximum accuracy of transthoracic bioimpedance was higher than that of heart rate variability, both contributed to the even higher accuracy of the model that combined them. We hypothesized that acute decompensated heart failure participants at the time of admission (baseline group) would have significantly lower resistances than participants in the control and acute decompensated heart failure discharge groups. Our results showed statistically significantly lower *R_0_* and *R_0_ – R_∞_* resistances in the baseline group (mean 27 Ω, SD 13 Ω; mean 10 Ω, SD 6 Ω, respectively) than those in the control group (mean 38, SD 11 Ω; mean 17, SD 6.1 Ω, respectively), with *P* values of .006 and .001, respectively. This suggests that participants in the baseline group had higher fluid volumes retained in the lungs than participants in the control group did. Moreover, the same parameters *R_0_* and *R_0_ – R_∞_* for discharge participants (mean 34, SD 17 Ω; mean 14, SD 9 Ω, respectively) were higher than those for the baseline participants. However, this difference did not reach statistical significance (*P*=.99; *P*=.57, respectively). Since predischarge assessments could not be performed in all participants, our findings may be attributable to a relatively small sample size. Alternatively, significant variability in the amount of intrathoracic fluid remaining before discharge may also explain our findings.

Bioimpedance is a proven biomarker of acute decompensated heart failure. Our group previously performed a clinical study of 106 hospitalized patients discharged after an admission for acute decompensated heart failure. Participants were sent home with a fluid accumulation vests and we determined that it was feasible to measure transthoracic bioimpedance on a daily basis [12]. We also demonstrated that a predictive algorithm analyzing daily bioimpedance measures achieved good performance for predicting recurrent acute decompensated heart failure [12]. Lindholm et al [22] also performed a longitudinal study including over 500,000 participants and determined that leg bioimpedance was inversely correlated with new-onset heart failure and that by combining the leg bioimpedance with clinical parameters such as age, sex, and history of myocardial infarction, accurate prediction of heart failure could be achieved. In another study [23] on participants with congenital heart disease, bioelectrical impedance correlated with heart failure severity. In contrast to these prior studies, we sought to evaluate the performance of intrathoracic bioimpedance measured using a novel dry electrode for detecting acute decompensated heart failure. We observed that participants hospitalized with acute decompensated heart failure had lower values of intrathoracic resistance due to higher intrathoracic fluid volume.

As for the heart rate variability, high-frequency components of heart rate variability (at admission: *P*=.02; at discharge: *P*=.13) and normalized high-frequency components of heart rate variability parameters (at admission: *P*=.003, at discharge: *P*=.02) were significantly higher in acute decompensated heart failure participants when compared to control participants without acute decompensated heart failure. This is possibly the result of more labored breathing exhibited by the participants with acute decompensated heart failure [41]. Although not statistically significant, we observed overall higher sympathetic activation in the acute decompensated heart failure participants, as evidenced by higher low-frequency components of heart rate variability (control: mean 3.5, SD 4.2; at admission: mean 19.3, SD 43.4, *P*=.06; at discharge: mean 19.2, SD 51.3 *P*=.09). The activation of the sympathetic nervous system is a known countermeasure of the body aiming to restore cardiac output in the case of heart failure [42]. Conversely, acute decompensated heart failure participants exhibited a significantly lower normalized low-frequency components of heart rate variability but only in the discharge group. This was produced by the highly elevated parasympathetic tone (high-frequency components of heart rate variability), which affected the computation of the normalized indices (normalized low-frequency components of heart rate variability and normalized high-frequency components of heart rate variability). These results corroborate the alteration of the autonomic nervous functions produced by acute decompensated heart failure and explain why the parameters of the autonomic function are valuable for detecting acute decompensated heart failure and its subsequent consequences.

In the machine learning classifications, *R_0_* was consistently chosen in most of the optimal models and was present in both the most accurate models for both classifications tested in this study (control/baseline/discharge and patients without fluid/patients with fluid classification). This is in agreement with the between-group statistical differences, in which this parameter was found to be the most sensitive to heart failure. Using the set of transthoracic bioimpedance parameters only, machine learning models were able to provide moderate classification accuracy for both types of classification: an accuracy of 68% was found for 3-class classification (control/baseline/discharge classification) model, and an accuracy of 82% was found for 2-class models (patients without fluid/patients with fluid classification), which are acceptable performances, considering that the bottom line accuracy for 3- and 2-class models are 33% and 50%, respectively. However, adding heart rate variability parameters (the model was trained with transthoracic bioimpedance and heart rate variability parameters together) further increased the accuracy of the models. The control/baseline/discharge classification, with 75% accuracy, was acceptable. Furthermore, 92% accuracy for classifying of patients without fluid and patients with fluid suggested the feasibility of such an algorithm to potentially detect the healthy condition (control group) or recovering (at least partially) of a patient from excess fluid accumulation. This model used parameters from transthoracic bioimpedance (extracellular resistance, intracellular resistance, cell membrane capacitance), as well as parameters from heart rate variability (low-frequency components, principal dynamic mode index of parasympathetic function, mean heart rate). The transthoracic bioimpedance parameters that were included are related to intra- and extracellular fluid, whereas the heart rate variability parameters are mostly related to the sympathetic activation. This finding is useful in developing in-home diagnostic tools that can detect or predict fluid accumulation in heart failure participants.

Statistical analysis and machine learning analysis showed similar results for a reduced set of features. For instance, extracellular resistance and low-frequency components of heart rate variability exhibited significant differences between non–heart failure (control) and heart failure groups (baseline and discharge), and these features were present in the most accurate model for fluid accumulation detection. However, other features including intracellular resistance, cell membrane capacitance, principal dynamic mode index of parasympathetic function, and mean heart rate did not exhibit significant differences between groups but were relevant for improving accuracy of the machine learning algorithms.

### Limitations

As for the limitations of the study, many recordings were not usable, mostly in the acute decompensated heart failure group. This is related to technical issues with the fluid accumulation vests, which can be partially attributed to the carbon black–PDMS electrodes. From the 28 participants with acute decompensated heart failure, we obtained reliable measures from only 23 participants at baseline and from 17 participants at discharge. We obtained data from both baseline and discharge for only 12 participants. Even in the control group, we collected usable data from only 32 out of the 50 participants. In some instances, applying a layer of hydrating lotion helped with data collection. This limitation could potentially diminish the clinical use of the device and must be addressed in the near future. A more robust hardware design, tailored to match the impedance of the carbon black–PDMS electrodes, is a potential improvement. Configurations that enable collection of transthoracic bioimpedance data from several locations on the thorax could help the quality and usability of the data, as accumulation of fluid does not occur always in the same location. Furthermore, given the limited data set, we have reported leave-one-subject-out cross-validation accuracy, and the results cannot be interpreted as conclusive concerning the efficacy of the transthoracic bioimpedance device and features derived from it. Instead, the results can be interpreted as promising, based on the validation of the transthoracic bioimpedance and its associated features and machine learning. A larger testing data set is required for further evaluation of transthoracic bioimpedance to allow for more definite conclusions about its efficacy.

There are several potential clinical applications of transthoracic bioimpedance measurements in patients with heart failure. Wearable technologies such as fluid accumulation vests could allow for rapid point-of-care diagnostics that could be used in the emergency setting to help identify heart failure decompensation. In addition, fluid accumulation vest measurements in different clinical states such as decompensated heart failure, predischarge, and in outpatient setting, could be used to establish a profile for a given patient that could improve diagnostic certainty and guide treatment. Moreover, triaging medical severity is a necessary and time-consuming step of the patient care process, but this is often difficult due to limitations in both the number of available medical personnel and individual provider time.

The device and algorithm in this study can be used in a longitudinal study with patients with heart failure, extending monitoring into the home. The system could be used to monitor a patient’s fluid accumulation daily and generate early warnings of heart failure decompensation, provide guidance on therapeutic changes to improve quality of life, and reduce heart failure readmissions. Alternatively, the system can be used to monitor either the discharge readiness of a patient from the hospital or the home treatment regime effectiveness on the patient. Wearable sensors such as the fluid accumulation vest can potentially provide an ideal avenue for patient monitoring over time, allowing for rapid action in response to acute decompensation. Garments integrating vital sign sensors have been utilized in acute medical settings to monitor patients with high medical risk profiles [43]. In addition, wearable sensor-based systems for vital sign monitoring are well-received by both patients and nursing staff with regards to usability, further highlighting their potential role in clinical implementation [44].

### Conclusions

The main goal of this study was to evaluate the performance of biologically relevant parameters measured by a fluid accumulation vests with carbon black–PDMS dry electrodes. In our clinical study (SHIELD), transthoracic bioimpedance and heart rate variability parameters were considered for statistical analysis and discrimination between patients with nonacute decompensated heart failure and acute decompensated heart failure. As expected, our results show that among the 15 parameters, 2 (extracellular resistance and intracellular-extracellular difference in resistance) showed statistically significantly lower values (*P*=.006; *P*=.001, respectively), and 3 (tissue heterogeneity exponent, high-frequency components of heart rate variability, and normalized high-frequency components of heart rate variability) showed statistically significantly higher values (*P*=.01, *P*=.02, *P*=.003, respectively) for participants with acute decompensated heart failure at hospital admission than those for participants in the control group. A significant difference in the sympathetic control (assessed with the normalized low-frequency components, *P*=.01) was observed between acute decompensated heart failure participants at the time of discharge and the control participants. Transthoracic bioimpedance and heart rate variability exhibited promising results for classifying participants with excess intrathoracic fluid versus those with normal intrathoracic fluid. Further clinical studies will be undertaken to refine our approach and determine the optimal application of this monitoring technology in acute medical settings.

## Abbreviations

AF: atrial fibrillation
ECG: electrocardiography
ESRD: end-stage renal disease
HRV: heart rate variability
ICD: implantable cardioverter-defibrillator
PDMS: polydimethylsiloxane
